# Identification and Characterization of a PRDM14 Homolog in Japanese Flounder (*Paralichthys olivaceus*)

**DOI:** 10.3390/ijms16059097

**Published:** 2015-04-23

**Authors:** Lin Fan, Jiajun Jiang, Jinning Gao, Huayu Song, Jinxiang Liu, Likun Yang, Zan Li, Yan Chen, Quanqi Zhang, Xubo Wang

**Affiliations:** Key Laboratory of Marine Genetics and Breeding (Ocean University of China), Ministry of Education, Qingdao 266003, China; E-Mails: fanlin_lynne@sina.cn (L.F.); j15964239284@163.com (J.J.); gjn.1127@163.com (J.G.); song.huayu@163.com (H.S.); ljkmn911@outlook.com (J.L.); yanglikun0320@126.com (L.Y.); xiumei0210@163.com (Z.L.); gtpeng@163.com (Y.C.); qzhang@ouc.edu.cn (Q.Z.)

**Keywords:** Japanese flounder (*Paralichthys olivaceus*), *prdm14*, gene expression, promoter analysis, pluripotency, primordial germ cell (PGC)

## Abstract

PRDM14 is a PR (PRDI-BF1-RIZ1 homologous) domain protein with six zinc fingers and essential roles in genome-wide epigenetic reprogramming. This protein is required for the establishment of germ cells and the maintenance of the embryonic stem cell ground state. In this study, we cloned the full-length cDNA and genomic DNA of the *Paralichthys olivaceus prdm14* (*Po*-*prdm14*) gene and isolated the 5' regulatory region of *Po*-*prdm14* by whole-genome sequencing. Peptide sequence alignment, gene structure analysis, and phylogenetic analysis revealed that *Po*-PRDM14 was homologous to mammalian PRDM14. Results of real-time quantitative polymerase chain reaction amplification (RT-qPCR) and *in situ* hybridization (ISH) in embryos demonstrated that *Po*-*prdm14* was highly expressed between the morula and late gastrula stages, with its expression peaking in the early gastrula stage. Relatively low expression of *Po*-*prdm14* was observed in the other developmental stages. ISH of gonadal tissues revealed that the transcripts were located in the nucleus of the oocytes in the ovaries but only in the spermatogonia and not the spermatocytes in the testes. We also presume that the *Po*-*prdm14* transcription factor binding sites and their conserved binding region among vertebrates. The combined results suggest that *Po*-PRDM14 has a conserved function in teleosts and mammals.

## 1. Introduction

PRDM (PRDI-BF1-RIZ1 homologous domain-containing) family members are characterized on the basis of a conserved *N*-terminal PR domain and several *C*-terminal Krüppel-type zinc finger motifs. The PR domain was first reported as the PRDI-BF1-RIZ1 homologous domain [1], whose name was derived from the two members: PRDI-BF1 (positive regulatory domain I-binding factor 1) and RIZ1 (retinoblastoma-interacting zinc finger protein 1) [2,3]. To date, PRDI-BF1 is referred to as PRDM1 and RIZ1 as PRDM2 [4]. The additional PRDM family members were subsequently named. The PR domain has a 20%–30% amino-acid identity to the SET (suppressor of variegation 3–9, enhancer of zeste, and trithorax) domain, which is a conserved methyltransferase domain found in all known lysine histone methyltransferases (HMTs) that methylate histone tails [5,6,7,8]. PRDM family members act as direct HMTs (PRDM2, PRDM3, PRDM8, PRDM9, PRDM6, and PRDM16) [9,10,11,12,13,14] or recruit a suite of histone-modifying enzymes to target promoters [15]. Previous studies revealed that *prdm* genes expanded in vertebrates and *prdm7*/*prdm9* further duplicated in primates. The PRDM family in primate genomes consists of 17 putative orthologs (such that a single rodent co-ortholog corresponds to the primate pair formed by PRDM7 and PRDM9). By contrast, the PRDM family in rodents, birds, and amphibians contains 16 putative orthologs. Meanwhile, researchers only detected 15 orthologs in fish because they could not identify the PRDM17 ortholog, which was possibly because of the genome assembly used [16]. The above mentioned identified members of the PRDM family and their orthologs serve as potent regulators of important processes, such as cell development, cell fate specification, cell differentiation, and stem cell maintenance.

As a PRDM family member, PRDM14 has an *N*-terminal PR domain and six Krüppel-type zinc finger motifs. The PR domain and zinc fingers are highly conserved among vertebrates. In general, PRDM14 recruits genomic targets through the zinc finger domains. Previous studies identified that the PRDM14-bound regions contain the unique non-repetitive sequence 5'-GGTCTCTAA-3' in humans and mice [17,18]. This binding motif is distinct from the binding motifs of other PRDM family members, such as PRDM1 and PRDM9, indicating that each family member in the PRDM family has its unique binding sequence [19,20]. Otherwise, PRDM14-associated regions are usually far from promoters; less than 40% of the sites are located within 10 kb of the annotated transcriptional start sites (TSS), whereas more than 60% of the sites are located more than 10 kb from the TSS [17,18]. In addition, chromatin immunoprecipitation (ChIP)-sequencing revealed that 42.8% of the PRDM14-bound regions are also bound to NANOG, and 13.7% of this subset are also bound to OCT3/4. Other studies also claimed that PRDM14 co-localizes with SOX2. These results support the hypothesis that PRDM14 shares a substantial number of genomic targets with key transcription factors (TFs), particularly NANOG [17,21]. Moreover, PRDM14 directly regulates OCT3/4 (POU5F1) through its proximal enhancer [17,18,21]. A gain–of–function assay showed that PRDM14, in conjunction with OCT3/4, SOX2, and KLF4 can enhance the efficiency of reprogramming in human fibroblasts. Therefore, PRDM14 has an important role in the maintenance of hESC (human embryonic stem cells) identity and exemplifies a key TF for the reacquisition of pluripotency in human somatic cells [21]. PRDM14 plays an important role in the maintenance of ESC and iPSC (induced pluripotent stem cells) states [17,21,22,23], the reactivation of X chromosomes [22], the generation of PGCs [24], and the demethylation of defined human and mouse genes and loci [25,26,27]. PRDM14 in zebrafish is only involved in the axon growth of primary motor neurons [28]. The function of PRDM14 in other species remains to be investigated.

The Japanese flounder (*Paralichthys olivaceus*) is an important farmed marine fish, which shows morphological left-right asymmetry, such as one eye migrates to the opposite side (metamorphosis) during the larval stages. Similar to mammalian ESCs, fish ESCs can replicate indefinitely and differentiate into all three layers of the embryo. To date, some fish ESC lines have been established under a feeder-free condition from blastula-stage embryos [29,30,31,32,33]. Genetic resources can be protected with this method. The second method is reprogramming somatic cells to generate iPSCs, which is a novel strategy to obtain pluripotent stem cell lines. These iPSCs exhibit the morphology and growth properties of ESCs and express ESC marker genes [34]. When ES cells colonize germ cells in chimeras, transgenic animals with desirable genetic traits are generated and could be used for functional genomics studies or the improvement of commercial productivity [35]. Another method is using primordial germ cells (PGCs), the common source of oocytes and spermatozoa that carry the genetic resources of an organism. PRDM14 plays a significant role in the specification of PGCs, which could be used to protect genetic resources [24,36,37]. Given the significant roles of PRDM14 in ESCs, iPSCs and PGCs, in-depth research on the *Po-prdm14* gene is critical.

This work aimed to clone and characterize the *Po-prdm14* gene from Japanese flounder at the molecular level. RT-qPCR amplification, ISH, peptide comparison, phylogenetic analysis, gene structure analysis, and promoter sequence analysis were used to describe different aspects of the *Po-prdm14* gene. The results obtained in this study provide a foundation for further studies on the function of this gene.

## 2. Results

### 2.1. Molecular Characterization of Po-prdm14

#### 2.1.1. Cloning and Analysis of *Po*-*prdm14*

**Figure 1:**
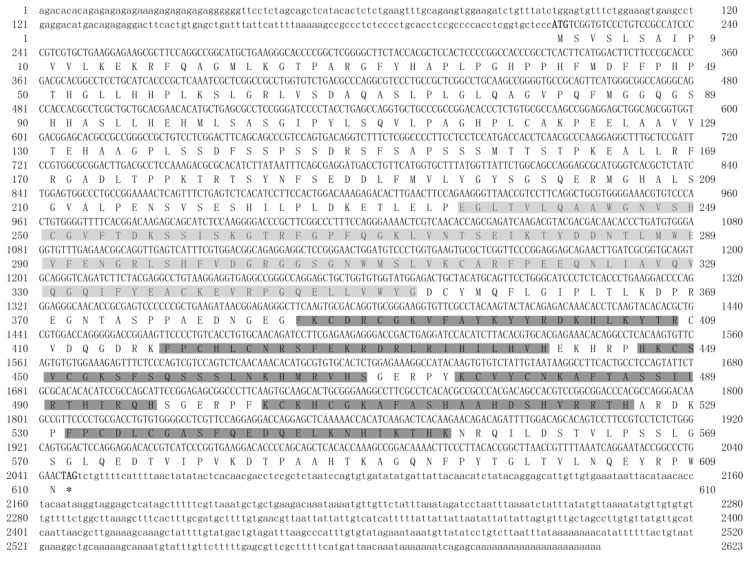
Nucleotide and deduced amino acid sequences of *Po-*PRDM14. The open reading frame (ORF) is denoted in uppercase letters, and the untranslated regions (UTRs) are denoted in lowercase. The deduced amino acids are indicated in the one-letter code and presented below the coding regions. The start and termination codons are highlighted in boldface. The SET domain and the six zinc fingers are shaded with light and dark gray boxes, respectively.

**Figure 2:**
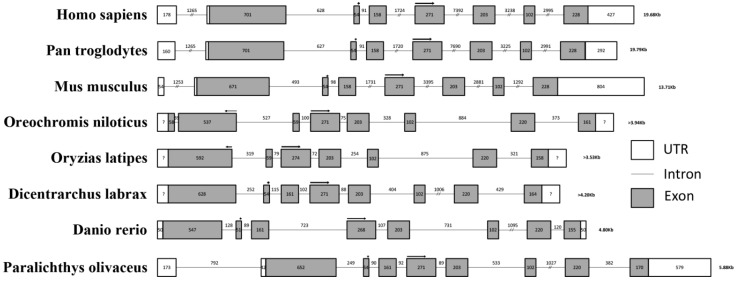
Gene structure analysis of *prdm14*. Comparison of the genomic organization of the *prdm14* gene between fish and mammals. Exons are shown in shaded boxes, and introns are underlined. The sizes of the primary transcripts and each section are indicated. The 5' and 3' UTRs are enclosed in boxes indicating their respective sizes. The length of the UTRs of *Oreochromis niloticus*, *Oryzias latipes*, and *Dicentrarchus labrax* are unknown, we use “?” to display the unknown sequences. All SET domain sequences are indicated by arrows.

The full-length cDNA sequence of *Po*-*prdm14* is 2623 bp long (KM624610) and has a 214 bp 5' UTR, a 576 bp 3' UTR, and a 1833 bp open reading frame (ORF) (Figure 1). *Po*-PRDM14 encodes 610 amino acid residues, and its estimated molecular mass is 67.76 kDa. Similar to other species, the predicted *Po*-PRDM14 peptide contains a SET domain at amino acid positions 235–352 (Figure 1). A comparison of cDNA and genomic sequences shows that *Po*-*prdm14* contains nine exons (Figure 2), which differs from the gene structure of most of the other known species. On the basis of gene structure analysis, the ORF structure of *Po*-*prdm14* is almost identical to that of *Dicentrarchus labrax prdm14* (*Dl-prdm14*) and *Danio rerio prdm14* (*Dr-prdm14*). In general, the 5' UTR sequences are located on the first exon and on part of the second exon.

**Figure 3:**
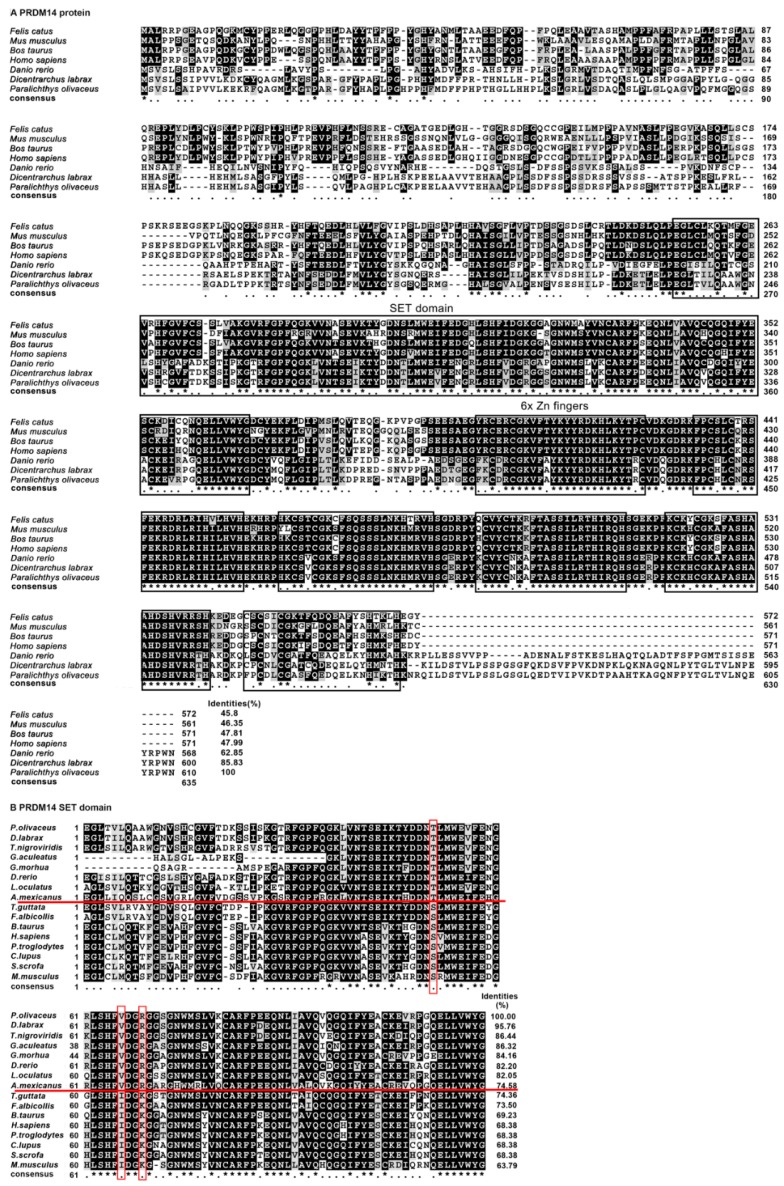
(**A**) Multiple alignment of full-length PRDM14 amino acid sequences in different species. The conserved SET domain is marked with a black frame. The identity scores relative to the Japanese flounder sequence are shown on the right; (**B**) SET domain protein sequence alignment of *Po*-PRDM14 with other vertebrate orthologs. Identity scores relative to the Japanese flounder SET domain sequence are shown on the right. The alignment is generated with the CLUSTALW and shaded with BOXSHADE 3.21. Identical (*) and similar (.) amino acid residues are indicated. The conserved positions in teleosts are marked with a red frame. The fish and tetrapod species are separated by a red line.

#### 2.1.2. Homology Analysis of *Po*-PRDM14

The alignment of the deduced amino acid sequences of *Po*-PRDM14 and other known PRDM14 orthologs was performed using ClustalX2.1 (Figure 3A). The PR domain and the zinc finger arrays of PRDM14 were highly conserved among the vertebrates, whereas the N-terminal region was divergent. Among the included species, *Po*-PRDM14 had the highest identity at 85.83% with *Dl*-PRDM14. Moreover, the SET domain alignment revealed an extremely high identity ranging from 63.79% to 95.76% (Figure 3B). Importantly, much higher sequence identity was observed among fish (>74.58%). Three conserved positions were identified from the alignment: threonine (T51), valine (V66), and arginine (R69) were in fish, whereas serine (S51), isoleucine (I66), lysine (K69) were in tetrapods.

**Figure 4:**
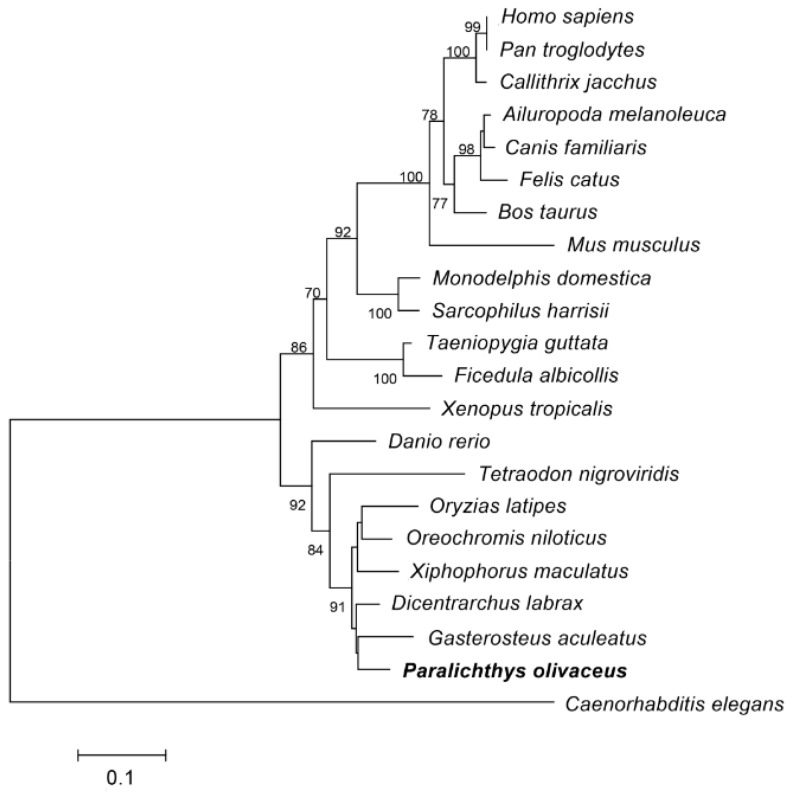
Phylogenetic analysis of *Po*-PRDM14 in comparison with PRDM14 or PRDM14-like proteins in other representative vertebrates based on their amino acid sequences. *Po*-PRDM14 is marked with bold fonts. The phylogenetic tree was constructed by MEGA from the Poisson correction distance via the neighbor-joining method with 1000 bootstrap replicates. The PRDM14 sequence of *Caenorhabditis elegans* served as the outgroup. The scale bar represents 0.1. The Ensembl IDs and accession numbers are as follows: *Homo sapiens*, Q9GZV8; *Pan troglodytes*, H2QWA1; *Callithrix jacchus*, F7I6N5; *Ailyropoda melanoleuca*, D2H9L4; *Canis familiaris*, E2QUL5; *Felis catus*, M3VWM4; *Bos taurus*, E1BCK6; *Mus musculus*, E9Q3T6; *Monodelphis domestica*, F6Y7L0; *Sarcophilus harrisii*, G3WS31; *Taeniopygia guttata*, H3CRY6; *Ficedula albicollis*, U3K0W4; *Xenopus tropicalis*, F6TMC6; *Danio rerio*, F1QWQ9; *Tetraodon nigroviridis*, F1QWQ9; *Oryzias latipes*, H2LVN9; *Oreochromis niloticus*, I3K9W9; *Xiphophorus maculatus*, M4A721; *Dicentrarchus labrax*, E6ZIN6; *Gasterosteus aculeatus*, G3NEF3; *Paralichthys olivaceus*, KM624610; *Caenorhabditis elegans*, Q20537.

The phylogenetic analysis of *Po*-PRDM14 in Figure 4 showed that the PRDM14 proteins could be grouped into two distinct clades: the fish and tetrapod subgroups, with above 70% bootstrap support. *Po*-PRDM14 clustered together with the teleost fish PRDM14 homologs.

### 2.2. Genomic Analysis of Po-prdm14

**Figure 5:**
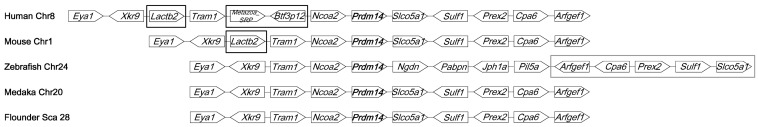
Chromosome localization analysis of *prdm14*. Chromosome synteny of fish and mammalian *prdm14* genes. The differences among human, mouse, and zebrafish sequences were marked with black and grey frames. Chr, chromosome, Sca, scaffold.

On the basis of the most recent *P. olivaceus* whole-genome sequence, *Po*-*prdm14* was located on Scaffold 28. Chromosomal syntenic relationship analysis revealed that *prdm14* was generally flanked by *Ncoa2* and *Slco5a1*, whereas *Danio rerio* probably underwent chromosomal rearrangement during its evolution (Figure 5). Moreover, given the arrangement of the neighboring genes of *prdm14*, the *Po*-*prdm14*-containing region was mostly syntenic to the *Ol-prdm14* gene from *Oryzias latipes*. This result agreed with previous studies [38].

**Figure 6:**
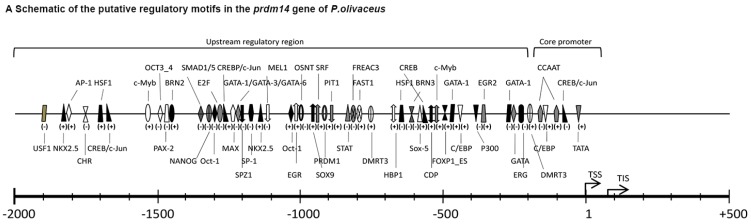

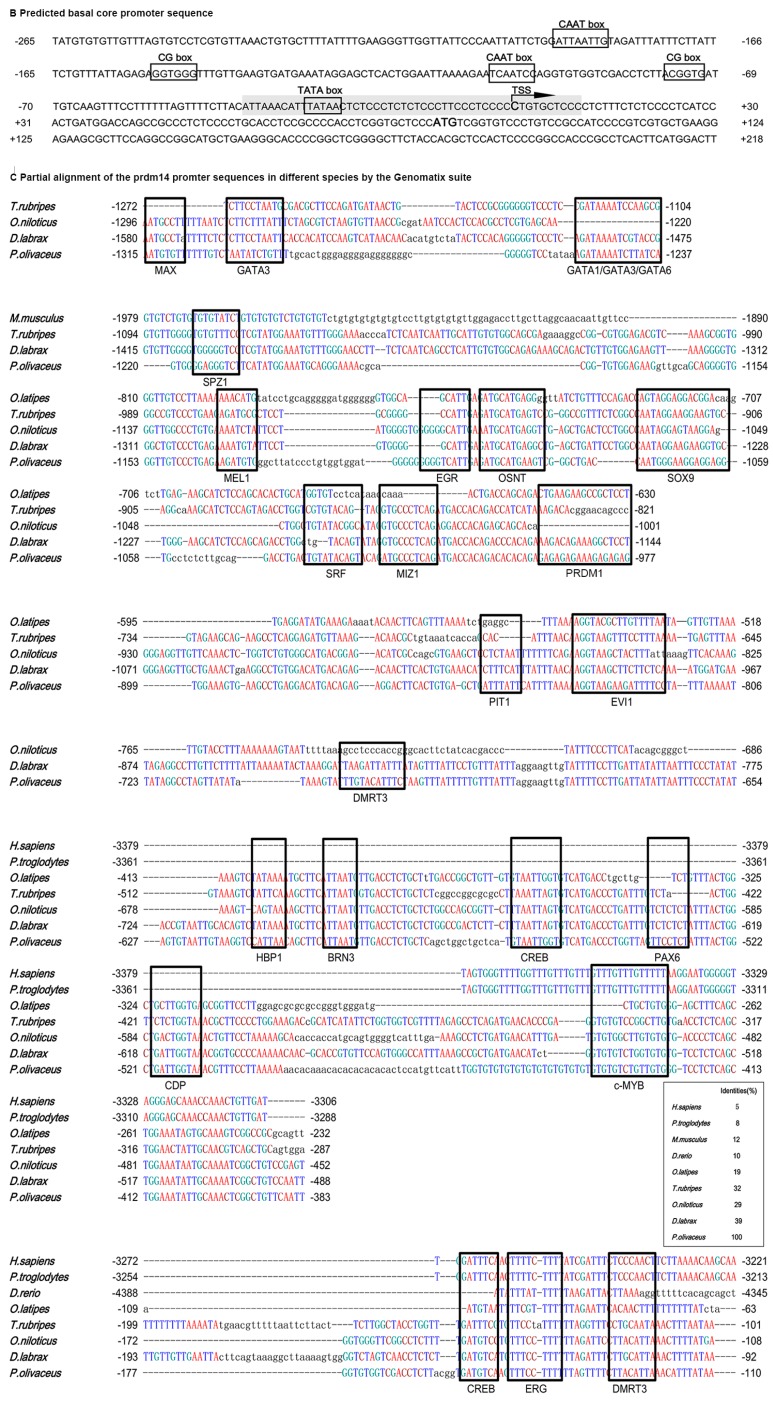
*Po-prdm14* promoter sequence features. (**A**) Schematic of the putative regulatory motifs in the *Po-prdm14* gene. The potential transcriptional start site (TSS; +1) was predicted by the NNPP program; the prediction score was greater than 0.90. The scale is given, and “±” indicates the TF-binding strand. TSS, Transcription start site; TIS, Translational initiation site; The full names of the potential TF binding sites are provided as follows; USF1: Upstream stimulating factor 1; NKX2.5: NKX homeodomain factors; AP-1: Activator protein 1; HSF1: Heat shock factor 1; SP-1: Specificity protein 1; CHR: Cell cycle gene homology region (CDE/CHR tandem elements regulate cell cycle dependent repression); CREB: cAMP-responsive element binding proteins; c-MYB: Cellular and viral myb-like transcriptional regulators; PAX-2: Paired box protein 2; SRF: Serum response factor; CDP: Transcriptional repressor CDP; FREAC3: Fork head related activator-3 (FOXC1); PRDM1: PR domain containing 1; Motif composed of binding sites for pluripotency or stem cell factors: OCT3/4, Nanog, OSNT, FOXP1_ES. OCT3/4: POU domain, class 5, transcription factor 1; OSNT: Composed binding site for Oct3/4, Sox2, Nanog, Tcf3 (Tcf7l1) and Sall4b in pluripotent cells; NANOG: Homeobox transcription factor Nanog; FOXP1_ES: alternative splicing variant of FOXP1, activated in ESCs; MAX: F-box leucine-rich repeat family protein; BRN2: Brn POU domain factor 2; STAT: Signal transducer and activator of transcription; DMRT3: Doublesex and mab-3 related transcription factor 3; SMAD1/5: vertebrate SMAD family of transcription factors; HBP1: HMG box-containing protein 1. EGR: Early growth response 1; GATA binding factors: GATA1, GATA3, GATA6. PIT: GHF-1 pituitary specific pou domain transcription factor. E2F: myc activator/cell cycle regulator, involved in cell cycle regulation, interacts with Rb p107 protein; OCT-1: Octamer-binding factor 1; SPZ1: Spermatogenic Zip 1 transcription factor; ERG: v-ets erythroblastosis virus E26 oncogene homolog; MEL: (MDS1/EVI1-like gene (1) DNA-binding domain. SOX/SRY-sex/testis determinig and related HMG box factors: SOX5, SOX9 and HBP1. SOX5: SRY-related HMG-box gene 5. SOX9: SRY-related HMG-box gene 9; (**B**) Predicted basal core promoter sequence. The promoter region predicted by NNPP is shaded. The core promoter elements are boxed and specified. The TSS and start codon are highlighted in boldface; and (**C**) Partial speculative alignment of the *Po-prdm14* promoter sequences in different species by the Genomatix suite. Selected conserved potential TF binding sites are indicated with black frames. The genomic sequences of different species were obtained from the ENSEMBL genome database and numbered with start codon ATG as +1. The Gene IDs are as followed: *H. sapiens*, ENST00000276594; *P. trogoldytes*, ENSPTRT00000037652; *M. musculus*, ENSMUST00000047577; *D. rerio*, ENSDART00000123674; *O. latipes*, ENSORLT00000010173; *T. rubripes*, ENSTRUT00000029807; *O. niloticus*, ENSONIT00000017929; *D. labtax*, CBN81920. The identity scores relative to the Japanese flounder sequence are shown on the right.

The potential TF binding sites within the 5' regulatory region of *Po*-*prdm14* revealed that multiple putative TFs bind to the said region in *Po*-*prdm14*, including heat shock factors 1 (HSF1); POU domain, class 5 transcription factor 1 (Oct3/4); the composite binding site for Oct3/4, Sox2, Nanog, Tcf3 (Tcf7l1), and Sall4b in pluripotent cells (ONST); SMAD1/5; PR domain containing 1 (PRDM1); activator protein-1 (AP-1); and specificity protein 1 (SP-1) (Figure 6A). A TATA box was located at −26 to −30 bp. Two GC boxes were separately located from −71 to −75 bp and −145 to −150 bp. Two CAAT boxes were separately located from −96 to −102 bp and −183 to −189 bp upstream of the transcription start site. These putative components comprised a putative basal core promoter (Figure 6B).

Considering that the TSSs of some species were not known, we had to designate the translational initiation site (ATG) as +1 (Figure 6C). The *Po*-*prdm14* promoter sequence had a higher similarity to those of sea bass (39%) and fugu (32%) than to those of other vertebrates. A total of 23 putative conserved regions were identified in the alignment of teleost homologs. One region was also conserved in the similar upstream region of the mouse homolog, whereas four regions were conserved among humans, chimpanzees and teleosts, but not rodents.

### 2.3. Expression and ISH of Po-prdm14 in Different Developmental Stages

**Figure 7:**
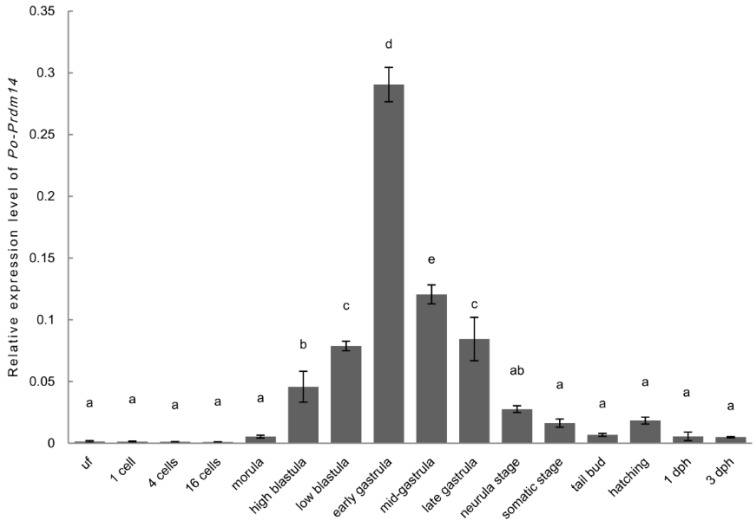
Relative expression of *Po-prdm14* during embryo development from the unfertilized egg to the hatching stage in *P. olivaceus*. The relative expression variance is presented as a ratio (the amounts of *Po-prdm14* mRNA normalized to the values of the corresponding reference genes). Data are shown as the mean ± SEM (*n* = 3; *p* < 0.05). Groups marked with the same letters are not statistically different.

The RT-qPCR results showed that *Po*-*prdm14* mRNA-positive signals were almost undetectable before the morula stage. As embryonic development progressed, the amount of *Po*-*prdm14* transcripts rapidly increased, and peaked at early gastrula stage before they suddenly decreased from the mid-gastrula stage onward. The amount of *Po*-*prdm14* transcripts were greatly reduced at the end of the gastrulation stage. Subsequently, the *Po*-*prdm14* transcripts were maintained at very low levels, with only a slight increase during the hatching stage (Figure 7).

**Figure 8:**
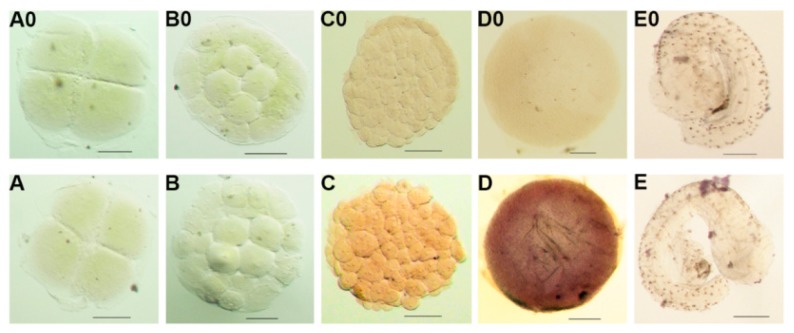
Expression of *Po-prdm14* mRNA during early embryogenesis by whole mount *in situ* hybridization. The positive cells with anti-sense probe hybridization were stained pink or purple (**A**–**E**), whereas the negative controls with sense probe hybridization remained unstained (A0-E0) at the (**A**) 4-cell; (**B**) 16-32 cell; (**C**) morula; (**D**) early gastrula, and (**E**) tail bud stages. Scale bars, 100 µm. ISH results revealed that the *Po-prdm14* mRNA-positive signals were the strongest in the early-gastrula stage and present in all blastomeres. Similarly, no signals of *Po-prdm14* mRNA were detected in the 4-cell, 16-cell, morula and tailbud stages.

Consistent with the RT-qPCR results, ISH results revealed that the *Po-prdm14* mRNA-positive signals were the strongest in the early-gastrula stage and present in all blastomeres. Similarly, no signals of *Po-prdm14* mRNA were detected in the 4-cell, 16-cell, morula and tailbud stages (Figure 8).

### 2.4. ISH of Po-prdm14 in the Gonad Sections

**Figure 9:**
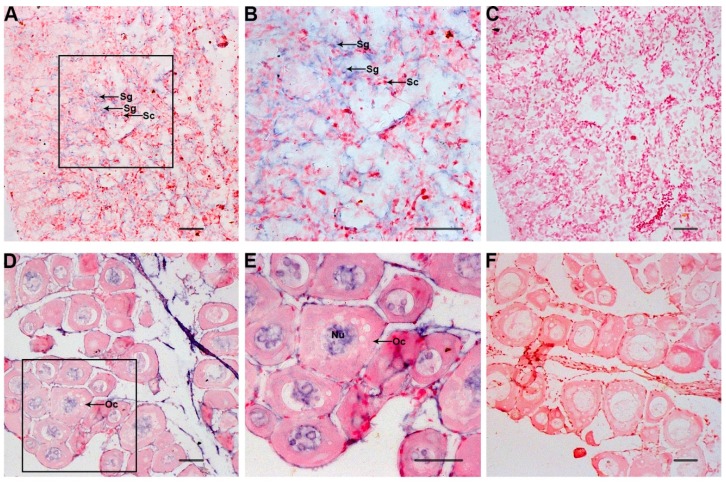
Expression of *Po-prdm14* mRNA in the gonads analyzed by *in situ* hybridization and histology. The *Po-prdm14* mRNA-positive cells (**A**,**B**,**D**,**E**) were stained as purple or blue, whereas the negative control with sense probe hybridization (**C**,**F**) was unstained. The box indicates the area magnified in the next frame (**A**,**C**,**D**,**F**: 20×; **B**,**E**: 40×). The *Po-prdm14* mRNA transcripts were observed in the spermatogonia, but no signals were detected in the spermatocytes in the testis (**A**,**B**). Noticeable positive signals were exhibited in the nucleus of oocytes in ovary (**D**,**E**). Sg, Spermatogonia; Sc, Spermatocytes; Oc, Oocytes; Nu, Nucleus. Scale bars, 25 μm.

The distribution of *Po-prdm14* transcripts in the gonadal sections was analyzed by ISH (Figure 9). Histological observation of the testis was performed at the telophase of Stage III, which mainly consists of primary and secondary spermatocytes, with spermatogonia sporadically situated adjacent to the interstitial compartment and at the intertubular location of the spermatogenic tubules. *Po-prdm14* expression was only found in the spermatogonia, whereas almost no signals were detected in the primary and secondary spermatocyte. Histological observation of the ovary was performed at the telophase of Stage II, which mainly consists of oocytes. In Stages II and III, oogonia were not found in these sections possibly due to their rare number. The results showed that *Po-prdm14* was located in the nucleus of the oocytes during oogenesis (Figure 9).

## 3. Discussion

Po-PRDM14, particularly its conserved PR domain and zinc fingers, shared high amino acid sequence identity with other species (Figure 3), which was consistent with previous studies [39]. Therefore, these two conserved domains might have important functions in organisms. The PR domain diverged from the SET domain, which defines a large group of HMTs. Although most of PRDM family members have not demonstrated such activity, PRDM14 can recruit a suite of histone-modifying enzymes to target promoters [25]. Zinc fingers are indispensable for recruitment to genomic targets (sequence-specific). Interestingly, five of the six zinc fingers found in *Po*-PRDM14 were highly conserved, whereas one was divergent. This result coincided with the conclusions of Ng *et al.* [40]. Phylogenetic analysis demonstrated that all teleosts grouped together in a subgroup, whereas the primates, rodents, birds and amphibians were assembled into another subgroup, namely, the tetrapod subgroup. Phylogenetic analysis demonstrated that *Po*-PRDM14 belonged to the teleost subgroup (Figure 4). The phylogenetic tree showed that *Po*-PRDM14 clustered with the PRDM14 homolog from *Gasterosteus aculeatus*, which were then clustered with the homolog from *Dicentrarchus labrax*. These three PRDM14 homologs formed their own clade. On the basis of the protein sequences, the conserved and characteristic domains, and the phylogenetic analysis, the Po-PRDM14 was demonstrated to be homologous to mammalian PRDM14.

We compared the *Po-prdm14* gene structures and other vertebrates (Figure 2). Since it was not necessary to consider the UTR sequences in this study, we only focus on the ORF sequences. The ORF sequences of mammals were highly conserved. Each had seven exons, with a SET domain between the second exon at 54 bp and the fourth exon at 271 bp. By contrast the number of exons and the position of the SET domain were not highly conserved in teleosts. In *Oreochromis niloticus*, the ORF of *prdm14* had eight exons, and its SET domain was located between the second exon at 537 bp and the fourth exon at 271 bp. In *Oryzias latipes*, the ORF had seven exons, and the SET domain located between the first exon numbered 592 bp and the third exon numbered 274 bp. In *Dr-prdm14*, the ORF had eight exons, and the SET domain located between the second exon numbered 51 bp and the fourth exon numbered 268 bp. In *Dl*-*prdm14* and *Po*-*prdm14*, the ORF had eight exons and the SET domain located between the second exon numbered 54 bp and the fourth exon numbered 271 bp. The disparity between the sequences of teleost fish and mammals may be explained by previous genomic studies. Teleost fishes comprise the largest and most diverse group of vertebrates [41]. Whole-genome duplication (WGD) provides the necessary genetic raw material for teleost radiation; subsequently after WGD, the rearrangement rate increases [42,43]. Therefore, the WGD may account for the rearrangement of the teleost genome. In addition, recent comparative genomic studies have revealed that irrespective of their duplication status, teleost genomes experienced frequent gene-linkage disruptions as compared with other vertebrates; the protein-coding sequences in teleosts were also evolving faster than those in mammals [41]. The *prdm14* sequences among teleosts did not coincide with each other very well, whereas the mammalian sequences had the same number of exons, and their SET domain was located at the same position. This result was in line with the above-mentioned findings of previous studies.

The chromosome synteny of the *prdm14* gene was analyzed among the fish, mouse, and human genomes (Figure 5). The results revealed that the gene arrangement near *Po*-*prdm14* had the highest identity with *Ol*-*prdm14*. This result agreed with the above-mentioned phylogenetic analysis (Figure 4). Figure 4 shows that *D. rerio* separately clustered from other teleosts. Chromosome synteny analysis (Figure 5) showed that the genes around *Dr-prdm14* were significantly different from the other teleosts. Compared with *Po*-*prdm14*, insertion and inversion may have occurred in the *D. rerio* chromosome during its evolution. This result supports the phylogenetic analysis and confirms that the largest and most diverse group of vertebrate fish genomes experienced various changes during its transmutation. Moreover, during the evolution from fish to rodents and humans, gene insertion likewise occurred in the chromosome regions around the *prdm14* gene.

The promoter region regulates gene expression; TFs bind to this region to activate or suppress gene expression. Several groups have demonstrated the presence of binding sites on the *prdm14* promoter. For example Boyer *et al.* [44] found that OCT3/4, SOX2, and NANOG occupy at least 353 genes in human ES cells, and *prdm14* is one of the said genes. Although the significance of the *prdm14* promoter has been previously discovered, the systematic analysis of this promoter remains lacking to the best of our knowledge. In the present study, we predicted the TF binding sites of the *Po-prdm14* promoter with online prediction software, and predicted the TSS of *Po*-*prdm14*, and aligned the *Po*-*prdm14* promoter with those of other species. Based on our prediction, we expected TSS (+1) to be a cytosine (C) residue located 85 bp upstream from the start codon ATG. Consensus basal core promoter elements, such as the TATA-box, GC-box and CAAT-box, were assumed in their expected positions; these elements might comprise the core promoter of *Po*-*prdm14* (Figure 6A). The online prediction software was used to predict numerous TF binding sites with a high matrix weight, which varied from 0.900 to 1.000 (Figure 6B). These binding sites might suggest PRDM14 function in some respect, and could be divided into three parts:

**A**. Motif composed of putative binding sites for pluripotency or stem cell factors. Such factors include OCT3/4 (POU domain, class 5, transcription factor 1), OSNT (composed of binding sites for OCT3/4, SOX2, NANOG, TCF3 or TCF7l1, and SALL4b in pluripotent cells), NANOG (homeobox transcription factor NANOG), and FOXP1_ES (alternative splicing variant of FOXP1, which is activated in ESCs). Interestingly, PRDM14 co-localized with OCT3/4, SOX2, NANOG, and P300 [17,21] and bound to the OCT3/4 upstream regulatory region in mammals [21]. It is known that mammalian PRDM14 and NANOG can co-operate [17,21]. A previous study in Japanese flounder releaved that *Po-nanog* was maternally inherited, expressed highly before gastrula and expressed specific in gonads among adult tissues. The analysis in *Po-nanog* showed potential *Po*-PRDM14 binding sites on *Po-nanog* promoter [38]. In this study, we also predicted the NANOG binding site in the *Po*-*prdm14* promoter, which suggests that Po-NANOG and Po-PRDM14 might interact with each other to some extent.

**B**. Motif composed of putative binding sites for PGC-related or sexual development. Such predicted binding sites include DMRT3 (doublesex and mab-3-related transcription factor 3), SPZ1 (testis-specific bHLH-Zip transcription factors, spermatogenic Zip 1 transcription factor), HBP1 (HMG box-containing protein 1), SOX/SRY-sex/testis determining and other related HMG box factors: SOX5 (SRY-related HMG-box gene 5), SOX9 (SRY-related HMG-box gene 9), and PRDM1 (PR domain containing 1). The specification of PGCs occurs through the integration of three key events: repression of the somatic program, reacquisition of potential pluripotency, and the ensuing genome-wide epigenetic reprogramming; PRDM14 is critical to the last two events in mouse [24,39,45,46,47,48,49,50,51].

**C**. Motif composed of putative binding sites for regulation. Such prognostic binding sites include AP-1 (activator protein 1), SP-1 (specificity protein 1), CREB (cAMP-responsive element binding proteins), and GATA binding factors (GATA1, GATA3, GATA6).

The alignment of the *Po-prdm14* promoters and other vertebrates predicted that some binding sites, such as DMRT3, PRDM1, OSNT, and SOX9, were conserved among vertebrates, particularly among teleosts (Figure 6C). These predicted results provide fundamental knowledge to better understand the transcriptional regulation mechanism of *Po-prdm14*. The authenticity of our results should be verified by further experimentation.

The expression of *prdm14* is restricted to early embryonic tissues [26]. Therefore, our expression study of *Po*-*prdm14* mainly focused on different development stages (Figure 7). RT-qPCR of embryos revealed that *Po-prdm14* was barely expressed before the morula stage. This result contradicted the reported expression in mouse [26]. In mouse, *prdm14* is strongly expressed at the 2-cell stage and then deregulated at the 4-cell and 8-cell stages, before it is subsequently lost at the 16-cell stage. During the high-blastula stage, *Po*-*prdm14* expression rapidly increased and peaked at the early gastrula stage before it sharply decreased and maintained a very low level. This result coincided with previous reports using mouse embryos: *prdm14* has a unique expression pattern that is restricted to the morula, the ICM (Inner cell mass) of blastocysts, the early epiblast, and mESCs [17,18,24,26,48,52]. In addition, several studies in humans demonstrated that *prdm14* expression starts from the 8-cell stage and continues in the ICM [39,53,54]. Moreover, *prdm14* is expressed in hESCs, which are similar to mESCs [21,55,56].

Whole-mount in situ hybridization (WISH) showed that positive signals almost coincided with the RT-qPCR results (Figure 8). In addition, although RT-qPCR had very low expression in the early cleavage stage and the stages after late gastrulation, we could not find positive signals in the ISH results. This contradiction might be explained by the lower sensitivity of ISH than RT-qPCR. Subsequently *Po-prdm14* mRNA-positive signals were the strongest in the early-gastrula and present in all blastomeres. While in zebrafish, *Dr-prdm14* gene was maternally inherited. Sun *et al*. displayed the expression levels of *Dr-prdm14* in embryos at 2-cell stage, 64-cell stage and blastula stage with WISH analysis, and the result revealed high *Dr-prdm14* expression in these stages [7]. Liu *et al*. via WISH showed that *Dr-prdm14* expressed in early-gastrula, late-gastrula, bud stage, and in neuron precursors among somite stages [28]. Based on the two studies, *Dr*-prdm14 might express in almost all stages through zebrafish development and had important roles in the neural development in zebrafish. However, the expression pattern of *Po-prdm14* and *Mus musculus* (*Mm-prdm14*) differed from that of *Dr-prdm14*, as they specifically expressed in several stages. But through WISH technology we did not detect the *Po-prdm14* in neural expression. One possibility might be the functions of PRDM14 homologues were not strictly conserved despite their conserved protein sequences.

The testis samples for ISH analysis in this study were approximately nine months old and the ovary samples for ISH analysis in this study were approximately one and a half years old because the ovary development occurs later than testis development. The gonads were immature and had more gonocytes. In the ISH of testis sections, the signals were only found in the spermatogonia, and almost no signals were detected in the primary and secondary spermatocytes. In the ovary, the *Po-prdm14* gene was expressed in the nucleus of oocytes (Figure 9). These results might suggest that the *Po-prdm14* gene participated in the development of gonad in Japanese flounder.

*Prdm14* is a newly discovered gene. To date, most studies have focused on mammals [15,18,21,27,55,57,58] and zebrafish [28]. Only a few studies involved non-model teleosts. To address this knowledge gap, we report the full-length cDNA sequence and the genomic DNA sequence of *Po-prdm14*. Based on our analyses of protein alignment, phylogenetics, gene structure, and chromosomal synteny, we conclude that *Po*-*prdm14* encodes a *prdm14* ortholog. Several predicted potential TFs suggest their possible influence on *Po*-PRDM14 expression in an active or negative manner. RT-qPCR and WISH analyses of *Po*-*prdm14* demonstrated that its expression rapidly increased at the high blastula stage, peaked at the early gastrula stage, and subsequently decreased. The ISH of tissue sections revealed that the *Po-prdm14* transcripts were located in the nucleus of the oocytes in the ovary, as well as in the spermatogonia but not in the spermatocytes in the testis. To the best of our knowledge, this study is the first to identify and characterize the *Po-prdm14* gene in marine fish. This study provides a foundation for the in-depth study of the *Po-prdm14* gene and provides a potential method to improve the protection of genetic resources.

## 4. Experimental Section

### 4.1. Embryo and Fish Collection

Japanese flounder (*P. olivaceus*) samples were obtained from a commercial hatchery in Yantai, Shandong province, China. Fertilized eggs were obtained by artificial fertilization and were incubated at 17 ± 1 °C in sterile sea water with an open recirculation water system and sufficient air supply. The different embryonic stages were observed under a stereomicroscope. Three pools of samples at embryonic stage (14 embryonic stages: Unfertilized egg, 1-cell, 4-cell, 16-cell, morula, high blastula, low blastula, early gastrula, mid-gastrula, late gastrula, neurula, somite, tail bud, and hatching stages) and post-embryonic stages (two juvenile stages, 1 and 3 dph (days post-hatching), were separately collected from mixed families with a nylon net (100-mesh). Embryos and larvae were immersed in 1 mL of RNAwait liquid (Solarbio, Shanghai, China) overnight at 4 °C and then stored at −80 °C until further use. The staged embryos and gonads (ovary and testis) for ISH were washed twice by PBS (in DEPC) and then fixed in 4% paraformaldehyde in PBS overnight at 4 °C. After removing the envelopes, the embryos were dehydrated in a gradient series of increasing methanol concentration and stored in 100% methanol at −20 °C. Animal experiments were performed according to the Regulations for the Administration of Affairs Concerning Experimental Animals (China, 1988). The procedures were also approved by College of Marine Life, Ocean University of China (Qingdao, China).

### 4.2. RNA and Genomic DNA Extraction

Total RNA was separately isolated from the embryos using Trizol reagent (Invitrogen, Carlsbad, CA, USA) in accordance the manufacturer’s instructions [59]. The extracted total RNA was treated with DNaseI (Takara, Dalian, China) to remove DNA contamination and verified by PCR with β-*actin*-gene specific primers. First-strand cDNA synthesis was performed with 1 μg of total RNA and the PrimeScript™ RT reagent kit with gDNA Eraser (Takara, Dalian, China) following the manufacturer’s instructions. Genomic DNA was extracted from embryos via the phenol–chloroform method, precipitated with isopropanol, washed with 70% ethanol and 100% ethanol, and then dissolved in TE buffer [59]. The quality and quantity of the total RNA and genomic DNA were evaluated by 1.5% agarose gel electrophoresis and measured with a Nanophotometer Pearl apparatus (Implen GmbH, Munich, Germany).

### 4.3. Cloning and Sequencing of the Po-prdm14 Gene

A scaffold with the highest identity with the *Dicentrarchus labrax prdm14* (*Dl-prdm14*) gene was obtained from the whole-genome sequence of *P. olivaceus* as constructed by our laboratory (unpublished). After a BLAST query of both sequences on Spidey (http://www.ncbi.nlm.nih.gov/IEB/Research/Ostell/Spidey/), the general distribution of introns and exons in *Po-prdm14* gene was acquired and found to be similar to those of other known species. From this information, two pairs of primers (Table 1) were designed to obtain partial fragments of the gene (*prdm14*-core1 and *prdm14*-core2). These partial fragments had an overlap that could aid the assembly of the larger central region of the full-length *Po-prdm14* gene. The remaining unknown cDNA region was sequenced by 5' and 3' rapid amplification of cDNA ends (RACE) reactions with the SMART™ RACE cDNA Amplification kit (Clontech, Dalian, China) in accordance with the manufacturer’s protocol with RACE primers (*prdm14*-3' RACE-1 and -2 as well as *prdm14*-5' RACE-1, -2 and -3) (Table 1). On the basis of the assembled whole-length cDNA sequence, the specific primer pairs (*prdm14*-full long) were designed to verify the complete open reading frame (ORF). The genomic DNA sequence was obtained via amplification with three pairs of primers (*prdm14*-DNA1, −2 and −3).

**Table 1:** Primers used in this study.

Primers	Sequence (5'–3')	Usage
*prdm14*-core1-Fw	ATGGGTCACGCTCTATCTGG	Core fragment PCR
*prdm14*-core1-Rv	ATCCTCAGTCGGTCCCTCTT	Core fragment PCR
*prdm14*-core2-Fw	AGAAGAGGGACCGACTGAGG	Core fragment PCR
*prdm14*-core2-Rv	AGGACGGAAGGACTGTGCTG	Core fragment PCR
*prdm14*-ORF-Fw	CTCTGAAGTTTGCAGAAGTGG	Verify ORF
*prdm14*-ORF-Rv	AACGTTCACAAAAGCATCGC	Verify ORF
*prdm14*-DNA1-Fw	TTCCTCTATTTACTGGCTGATTGGT	Full-length PCR
*prdm14*-DNA1-Rv	CCGTTATCTGGAGAAGAGGACACAC	Full-length PCR
*prdm14*-DNA2-Fw	TTGAGAACGGCAGGTTGAGTC	Full-length PCR
*prdm14*-DNA2-Rv	GAAAGTCAAGGCGAATGATGCT	Full-length PCR
*prdm14*-DNA3-Fw	AGTCGTCCAGTCTCAACAAACACAT	Full-length PCR
*prdm14*-DNA3-Rv	GAGCGGAGGTCGTTGTGAGTAT	Full-length PCR
*prdm14*-promoter-Fw	ACACCCGCATCCACCTTC	Verify promoter
*prdm14*-promoter-Rv	AGTGGAGCGTGGTAGAAGC	Verify promoter
*prdm14*-3'RACE-1	CGAGAAGAGGGACCGACTGAG	3'RACE PCR
*prdm14*-3'RACE-2	ACACATCCGCCAGCATTCC	3'RACE PCR
*prdm14*-5'RACE-1	TGAGAGGGATGCCCAGGAAC	5'RACE PCR
*prdm14*-5'RACE-2	GAGATGCTGCTCTTGTCCGTG	5'RACE PCR
*prdm14*-5'RACE-3	ATGAAGTGAGGCGGGTGGC	5'RACE PCR
*prdm14*-RT-Fw	TCTGAGTCTCACATCCTTCCA	RT-qPCR
*prdm14*-RT-Rv	CTCAAACACCTCCCACATCAG	RT-qPCR
β-actin-Fw	GAGATGAAGCCCAGAGCAAGAG	Semi-RT-PCR
β-actin-Rv	CAGCTGTGGTGGTGAAGGAGTAG	Semi-RT-PCR
18S-RT-Fw	GGTAACGGGGAATCAGGGT	RT-qPCR
18S-RT-Rv	TGCCTTCCTTGGATGTGGT	RT-qPCR
UbcE-RT-Fw	TTACTGTCCATTTCCCCACTGAC	RT-qPCR
UbcE-RT-Rv	GACCACTGCGACCTCAAGATG	RT-qPCR
*prdm14*-ISH-Fw	ATTTAGGTGACACTATAGAAACAGCACAGTCCTTCCGTCCT	ISH-probe
*prdm14*-ISH-Rv	TAATACGACTCACTATAGGGTTTGCAGCCTTTCGTTACAGT	ISH-probe

A 4 kb upstream sequence in the *Po*-*prdm14* gene was found in the *P. olivaceus* genomic data. After a BLAST query in the GenBank database, the 1929 bp region in this sequence was predicted to be the *Po*-*prdm14* promoter sequence, whereas the other region belonged to the *Ncoa2* gene. A pair of specific primers (*prdm14*-promoter) was designed to verify the promoter sequence. All the amplified PCR products were separated by 1.5% agarose gel electrophoresis, purified with the Zymoclean Gel DNA Recovery kit, and then cloned into the PMD18-T vector (Takara, Dalian, China). Positive clones were screened by specific-primers and sequenced.

### 4.4. RT-qPCR Amplification

Primers for RT-qPCR were designed (Table 1); these primers spanned the introns to avoid the amplification of genomic DNA (*prdm14*-RT). A pre-experiment was performed to confirm the specificity of the cDNA PCR product. RT-qPCR was performed with a LightCycler480 apparatus in a 20 μL reaction volume containing 1× SYBR^®^ Premix Ex Taq™ II (Takara, Dalian, China), 0.2 μM of each primer, and 1 μL of dilute cDNA (10 ng/μL). The PCR profile used was 95 °C (5 min) for pre-incubation, followed by 45 cycles at 95 °C (15 s) and 60 °C (45 s). Melting curves were generated at the end of each run to verify the presence of a single product. A negative control (no templates) was included for the PCR run. The 18S ribosomal RNA (rRNA) (18S-RT) and the ubiquitin conjugating enzyme (UbcE) (UbcE-RT) sequences were selected to normalize the target gene levels via the standard curve method [38,60]. All samples were run in triplicate to decrease the manual error. The expression data were tested with SPSS 20.0 software. Significant differences between samples were analyzed by one-way ANOVA using Duncan’s test. Values with *p* < 0.05 were considered statistically significant. All data were expressed as the mean ± standard error of the mean (SEM).

### 4.5. ISH Analysis

The *Po*-*prdm14* embryos and tissues were analyzed by ISH with a 641 bp probe spanning the *C*-terminal coding region and the 3' UTR of the *Po*-*prdm14* cDNA (*prdm14*-ISH). DIG-labeled RNA sense and anti-sense probes were synthesized with a DIG RNA Labeling kit (SP6/T7; Roche, Mannheim, Germany) in accordance with the manufacturer’s instructions. The ISH of whole embryos and tissues was performed as described by Gao *et al*. [38].

### 4.6. Sequence Analysis

Homologous protein sequences were obtained through a BLAST search of the NCBI GenBank (http://blast.ncbi.nlm.nih.gov/Blast.cgi) and the EMBL Uniprot (http://www.uniprot.org/) database. Multiple sequence alignment of *Po-*PRDM14 and the PRDM14 sequences of other species was performed with ClustalX2.1. To evaluate the evolutionary relationship between the predicted *Po*-PRDM14 protein and other vertebrate PRDM14, we constructed a phylogenetic tree from the full-length peptide sequences with MEGA using the Poisson correction distance via the neighbor-joining method with 1000 bootstrap replicates. The PRDM14 sequence of *Caenorhabditis elegans* served as the outgroup. The conserved domains were predicted using the simple modular architecture research tool (SMART http://smart.embl-heidelberg.de/) [61,62], and an InterProScan5 sequence search (http://www.ebi.ac.uk/interpro/search/sequence-search) of the amino acid sequence of *Po-*PRDM14. Neural Network Promoter Prediction (NNPP, http://www.fruitfly.org/seq_tools/promoter.html) was used to predict the promoter element of *Po-prdm14*. Bioinformatics analyses of the promoter sequence and potential TF binding sites within the 5' regulatory region of *Po-prdm14* were performed with the online program MatInspector (http://www.genomatix.de/matinspector.html) [63], combined with Matrix Family Library Version 9.1 (Sep 2013), TFSEARCH (http://www.cbrc.jp/research/db/TFSEARCH.html), Match 1.0 (http://www.gene-regulation.com/cgi-bin/pub/programs/match/bin/match.cgi), Alibaba2.1 (http://www.gene-regulation.com/pub/programs/alibaba2/index.html), and TFBIND (http://tfbind.hgc.jp/) [64]. The Multiple Em for Motif Elicitation (MEME, http://meme.nbcr.net/meme/cgi-bin/meme.cgi) and Dialign software from the Genomatix suite (http://www.genomatix.de/cgi-bin/dialign/dialign.pl) were used to identify the conserved DNA motifs in the 5' regulatory region of *Po-*PRDM14 and other vertebrates.
